# Assessment of Right Ventricular Function in Patients With Acute Myocardial Infarction

**DOI:** 10.7759/cureus.22399

**Published:** 2022-02-20

**Authors:** Praveen K Baghel, Sunil K Tripathi, Althaf A Vahab, Puneet Aggarwal

**Affiliations:** 1 Department of Medicine, Shyam Shah Medical College, Rewa, IND; 2 Department of Cardiology, Shyam Shah Medical College, Rewa, IND; 3 Department of Cardiology, Atal Bihari Vajpayee Institute of Medical Sciences (ABVIMS) and Dr. Ram Manohar Lohia (RML) Hospital, New Delhi, IND

**Keywords:** tapse score, right ventricular function, inferior wall myocardial infarction, cardiovascular disease, anterior wall myocardial infarction

## Abstract

Introduction

We assessed the right ventricular function in patients with first acute anterior wall myocardial infarction (AWMI) and inferior wall myocardial infarction (IWMI) without associated right ventricular infarction and assessed the relation between right ventricular function and the in-hospital clinical outcomes.

Methods

The present study was an observational cross-sectional study, which enrolled a total of 200 patients with chest pain of <24 hours who were diagnosed with acute ST-segment elevation myocardial infarction (MI) for the first time. Echocardiography was performed with a special emphasis on the tricuspid annular plane systolic excursion (TAPSE) score. The in-hospital clinical outcomes include major adverse cardiac events (MACE), which refer to all-cause mortality, cardiovascular mortality, recurrent MI, heart failure, or stroke in patients with acute myocardial infarction (AMI).

Results

A total of 200 patients with AMI were enrolled in the study of which 66% were males. Of patients, 68% had AWMI and 32% had IWMI. Patients with AWMI had more right ventricular dysfunctional changes as compared to IWMI, as measured by TAPSE score (17.8 ± 4.64 mm vs. 19.87 ± 3.61; p = 0.01, respectively). The incidence of MACE was 27.9% in AWMI as compared to 12.5% in IWMI (41.9% vs. 18.75% had right ventricular dysfunction, respectively). The outcome of AWMI patients was poor as compared to IWMI patients, as measured by duration of hospital stay (9.5 ± 4.73 days and 6.6 ± 4.70 days, respectively) and mortality (17.64% in AWMI vs. 6.25% in IWMI). The patients of AMI with TAPSE score ≤18 mm, suggesting right ventricular dysfunction, had a higher rate of MACE compared to those with TAPSE score >18 mm, respectively, 36.23% vs. 12.2%.

Conclusion

From this study, it is concluded that AWMI results in a higher incidence of right ventricular dysfunction as compared to IWMI. Furthermore, patients with AMI with concomitant right ventricular dysfunction were found to have poorer outcomes in terms of longer duration of hospital stay, higher incidence of MACE, and higher mortality rate, as compared to patients of AMI without right ventricular dysfunction.

## Introduction

Cardiovascular disease (CVD) is one of the leading causes of death throughout the globe. In the 19th century, coronary artery disease (CAD) was more prevalent in developed countries; however, recent data suggest that it has become epidemic in developing countries too [1]. The data from the WHO state that India accounts for 1/5th of global deaths associated with CVD, specifically in the younger population. As per the Global Burden of Disease study, the Indian population reports 272 CVD deaths per 100,000 population compared to the global average of 235 CVD deaths [1,2].

Diabetes mellitus, hypertension, smoking, stress, obesity, sedentary lifestyle, vulnerable genetics, and a poor diet are the primary causes of CAD in the Indian population [3]. Acute coronary syndrome is the most common cause of morbidity and death in people with CAD and anterior wall myocardial infarction (AWMI) and inferior wall myocardial infarction (IWMI) are by far the leading cause of severe myocardial infarction (MI). Clinical and hemodynamic features of acute myocardial infarction (AMI) are, to a large extent, determined by the territory of the coronary artery involved. Right ventricular MI is frequently associated with inferoposterior wall MI. In recent years, right ventricular function in patients with CAD has received more importance. Right ventricular infarction more frequently causes low cardiac output and shock, which is an important cause of mortality [4-6].

Generally, the right ventricular function is preserved in AWMI; however, in recent years, many studies have shown right ventricular dysfunction in isolated AWMI. Furthermore, in patients with left ventricular dysfunction after MI, an important predictor of cardiovascular mortality is the right ventricular function [6,7]. There is a significant contribution of the interventricular septum apart from the right ventricular free wall in the function of the right ventricle and hence right ventricular dysfunction is expected in septal involvement in AWMI. Heightened sympathetic drive caused by AMI is another plausible mechanism that could affect the right ventricular function [7,8].

Left anterior descending coronary artery branches supply blood to the anterior wall of the right ventricle, and several autopsy studies have shown that acute left anterior descending coronary artery occlusion causes right ventricular infarction. But this relation has not been studied adequately so far [9,10]. Furthermore, echocardiography and its advanced techniques (2D echocardiography, M-mode, pulse Doppler, and tissue Doppler echocardiogram) are considered ideal tools for the assessment of right ventricular function. Thus, this study aims to evaluate the incidence of right ventricular dysfunction in AMI using echocardiography and also to study the clinical profile and the in-hospital complications in AMI patients with right ventricular dysfunction.

## Materials and methods

This was a cross-sectional, observational study conducted at a tertiary care center in India between January 2020 and June 2021. The study was approved by the institutional ethics committee and written informed consent was obtained from all the patients included in the study.

The study enrolled a total of 200 patients with chest pain of <24 hours who were diagnosed with acute ST-segment elevation MI (STEMI) for the first time. ST-segment elevation of >2 mm in two contiguous leads (V1-V6 for anterior wall MI) and ST-segment elevation of >1 mm in two contiguous leads (L2, L3, and aVF for IWMI) with cardiac enzyme elevation was considered as the criteria for infarction. The study excluded patients with prior MI, right ventricular infarction, valvular heart disease, congenital heart disease, left bundle branch block (LBBB) or paced rhythm, and cardiomyopathy.

Detailed history and clinical examinations of all the patients were done at the time of admission to the intensive coronary care unit (ICCU) as mentioned in the proforma. Routine laboratory investigations were also carried out. Continuous electrocardiography was performed followed by two-dimensional (2D) echocardiography using Vivid T8 cardiovascular ultrasound machine (GE Healthcare, Chicago, Illinois), with special emphasis on tricuspid annular plane systolic excursion (TAPSE) score, which measures longitudinal excursion between end-diastole and peak systole (<18 mm is right ventricular systolic dysfunction) [11]. The TAPSE score was assessed immediately upon admission during the baseline echocardiography assessment.

The clinical outcomes include major adverse cardiac events (MACE), which refer to all-cause mortality, cardiovascular mortality, recurrent MI, heart failure, or stroke in patients of AMI.

All the data were analyzed using SPSS version 22 (IBM Corp., Armonk, New York). All the differences in characteristics between the patient groups were evaluated using the chi-square test. The minimum significance value of the p-value was set at 0.05.

## Results

The study enrolled a total of 200 patients who were diagnosed with acute STEMI for the first time. The mean age of enrolled patients was 56.01 ± 12.67 years and the maximum number of patients (29%) belonged to the age group of 51-60 years. The youngest patient was a 19-year-old male and the oldest patient was an 84-year-old female. In this study, the number of male patients (62%) was considerably higher with a male to female ratio of 1.94:1. In both the age groups (<50 years and ≥50 years), males outnumbered females but the number of male cases was significantly higher in the age group <50 years (p = 0.0026). The most common presenting symptom of patients with AMI was chest pain (68%). The common risk factors associated with AMI were dyslipidemia (68%) and diabetes mellitus (62%). The common clinical sign of patients at the time of presentation was tachycardia (39%) and hypotension (32%). The baseline and demographic characteristics of all patients are outlined in Table 1.

**Table 1:** Baseline and demographic characteristics of all patients. BP: blood pressure; JVP: jugular venous pressure.

Characteristics	N (%)
Age, years (mean ± SD)	56.01 ± 12.67
15-30 years, n (%)	4 (2%)
30-40 years, n (%)	18 (9%)
41-50 years, n (%)	42 (21%)
51-60 years, n (%)	58 (29%)
61-70 years, n (%)	46 (23%)
>70 years, n (%)	32 (16%)
Gender, n (%)	
Male	132 (62%)
<50 years	52 (39%)
≥50 years	80 (61%)
Female	68 (38%)
<50 years	12 (18%)
≥50 years	56 (82%)
Risk factors, n (%)	
Tobacco use	82 (41%)
Alcohol	64 (32%)
Systemic hypertension	96 (48%)
Diabetes mellitus	124 (62%)
Dyslipidemia	88 (68%)
Symptoms, n (%)	
Chest pain	136 (68%)
Breathlessness	56 (28%)
Sweating	48 (24%)
Palpitation	24 (12%)
Syncope	16 (8%)
Clinical signs, n (%)	
Pallor	62 (31%)
Pulse <60/min	38 (24%)
Pulse >100/min	78 (39%)
BP <100 mmHg systolic	64 (32%)
Elevated JVP	24 (12%)
Pedal edema	36 (18%)

The clinical findings of all patients are depicted in Table 2. Electrocardiography revealed AWMI (septal wall MI + anteroseptal wall MI + high lateral wall MI + anterolateral wall MI + extensive anterior wall MI) in 136 cases and IWMI in 64 cases.

**Table 2:** Clinical findings of all patients. ECG: electrocardiography; TAPSE: tricuspid annular plane systolic excursion.

Variables	N = 200
ECG findings	
Septal wall	8 (4%)
Anteroseptal wall	64 (32%)
High lateral	6 (3%)
Anterolateral wall	42 (21%)
Extensive anterior wall	16 (8%)
Inferior wall	64 (32%)
Echocardiography (TAPSE score)	
>22 mm	50 (25%)
21-22 mm	41 (20.5%)
19-20 mm	40 (20%)
16-18 mm	44 (22%)
<16 mm	25 (12.5%)

Table 3 outlines heart rate, TAPSE score, and duration of hospital stay among AWMI and IWMI patients. The mean TAPSE score of patients with AWMI was 17.80 ± 4.64 mm, and in patients with IWMI, it was 19.87 ± 3.61 mm. This suggests that the patients with AWMI had greater right ventricular dysfunction as compared to that of patients with IWMI. The mean duration of hospital stay in patients with AWMI was 9.5 ± 4.73 days whereas, in IWMI patients, it was 6.6 ± 4.70 days (p = 0.0004). This suggests that patients with AWMI had a longer duration of hospital stay as compared to that of patients with IWMI. The patients with TAPSE score ≤18 mm mostly had >10 days of hospital stay (54.5% vs. 10.4%); however, patients with TAPSE score >18 mm had mostly ≤10 days of hospital stay (89.5% vs. 45.4%) (p = 0.0001).

**Table 3:** Heart rate, TAPSE score, and duration of hospital stay among AWMI and IWMI patients. AWMI: anterior wall myocardial infarction; IWMI: inferior wall myocardial infarction; TAPSE: tricuspid annular plane systolic excursion.

Variables	AWMI (n = 136)	IWMI (n = 64)	P-value
Heart rate			
<60 bpm (bradycardia)	16 (11.7%)	14 (21.9%)	0.001
60-100 bpm	50 (36.8%)	42 (65.6%)	
>100 bpm (tachycardia)	70 (51.5%)	8 (12.5%)	
TAPSE score, mm (mean ± SD)	17.80 ± 4.64	19.87 ± 3.61	0.008
>22 mm	26 (19.1%)	24 (37.5%)	
21-22 mm	25 (18.4%)	16 (25%)	
19-20 mm	28 (20.6%)	12 (18.7%)	
16-18 mm	36 (26.5%)	8 (12.5%)	
<16 mm	21 (15.4%)	4 (6.25%)	
Duration of hospital stay, days (mean ± SD)	9.5 ± 4.73	6.6 ± 4.70	0.0004
≤5 days	26 (19.1%)	30 (53.1%)	
6-10 days	56 (41.1%)	22 (31.2%)	
11-15 days	38 (28%)	8 (12.5%)	
>15 days	16 (11.8%)	4 (3.2%)	

All-cause mortality (17.6% vs. 6.2%) and MACE (27.9% vs. 7.6%, p = 0.001) were significantly higher in patients with AWMI compared to IWMI, respectively. Furthermore, all-cause mortality (26.1% vs. 7.6%) and MACE (36.2% vs. 12.2%, p = 0.001) were found to have higher incidence in patients with TAPSE ≤ 18 mm as compared to TAPSE > 18 mm. Tables 4, 5 outline adverse events among patients with AWMI vs. IWMI and ≤18 mm vs. >18 mm TAPSE score, respectively.

**Table 4:** Adverse events among patients with AWMI vs. IWMI. AWMI: anterior wall myocardial infarction; IWMI: inferior wall myocardial infarction; MI: myocardial infarction; MACE: major adverse cardiac events.

Adverse events	AWMI (n = 136)	IWMI (n = 64)	P-value
All-cause mortality	24 (17.6%)	4 (6.2%)	0.032
Cardiovascular mortality	22 (1.5%)	3 (4.7%)	0.065
Recurrent MI	16 (11.7%)	6 (9.4%)	0.059
Heart failure	32 (23.5%)	6 (9.4%)	0.002
Stroke	6 (4.4%)	00	0.013
MACE	38 (27.9%)	8 (12.5%)	0.034
No complications	98 (72%)	56 (87.5%)	0.345

**Table 5:** Adverse events among patients with ≤18 mm vs. >18 mm TAPSE scores. MI: myocardial infarction; MACE: major adverse cardiac events; TAPSE: tricuspid annular plane systolic excursion.

Adverse events	TAPSE ≤ 18 mm (n = 69)	TAPSE > 18 mm (n = 131)	P-value
All-cause mortality	18 (26.1%)	10 (7.6%)	0.023
Cardiovascular mortality	17 (24.6%)	9 (6.9%)	0.001
Recurrent MI	14 (20.3%)	8 (6.1%)	0.045
Heart failure	23 (33.3%)	15 (11.4%)	0.034
Stroke	4 (5.8%)	2 (1.5%)	0.004
MACE	25 (36.2%)	16 (12.2%)	0.030

## Discussion

The relevance of right ventricular dysfunction in the prognosis of patients with AMI has been underexplored so far. The present study aims to evaluate the right ventricular function in patients with AMI and to assess the relation of right ventricular function in the prognosis of these patients.

In this study, the maximum number of cases were from the age group of 51-60 years. The incidence of MI was observed to increase with age, and this could be due to the fact that atherosclerosis, which is associated with advancing age, is tuned by cumulative effects of lifestyle and lipid status. Indians have a higher risk of CAD at an earlier age because they have a higher prevalence of central obesity, decreased high-density lipoprotein (HDL) cholesterol, glucose intolerance, and hyperinsulinemia [12]. In the current study of 200 patients, males outnumbered females with a male to female ratio of 1.94:1. The male to female ratio in the age group of <50 years was 4.33:1 and in the age group of >50 years, it was 1.48:1. The higher incidence of infarction in males as compared to females at a younger age could be due to a higher prevalence of addiction habits like tobacco use, alcohol intake, and a more aggressive lifestyle. The protective role of estrogen in the reproductive age group of women may also have a role to play.

The prevalence of dyslipidemia in AMI patients was 68%, which was in line with other similar studies on the Indian population [13,14]. The higher prevalence of dyslipidemia in India could be attributed to the lack of awareness on the importance of exercise, inappropriate diet habits, decreased access to health care, and genetic predisposition [11]. TAPSE score is a technique for calculating the systolic excursion of the right ventricular annular segment in its longitudinal plane from an apical four-chamber image by passing an M-mode cursor through the tricuspid annulus at peak systole and measuring the annulus' longitudinal motion. The mean TAPSE score in patients of AWMI and IWMI was 17.80 ± 4.64 mm and 19.87 ± 3.61 mm, respectively. Also, 57/136 AWMI (41.9%) patients had a TAPSE score ≤18 mm as compared to 12/64 IWMI (18.75%) patients (p = 0.001). This suggests that contrary to the popular notion that right ventricular dysfunction is more common in IWMI, this study reveals that AWMI can result in a higher incidence of right ventricular dysfunction. This observation was in accordance with a study conducted by Aher et al. [15]. However, in a study conducted by Abdelsabour et al., it was observed that IWMI cases result in a higher incidence of right ventricular dysfunction as compared to AWMI [16]. This could be because cases of AMI with associated right ventricular MI were not excluded from the study sample.

Also, patients of AWMI were found to have a longer duration of hospital stay as compared to IWMI cases. The mean duration of hospital stay of AWMI and IWMI patients was 9.5 ± 4.73 days and 6.6 ± 4.70 days (p = 0.001), respectively. It was observed that 72% of patients with TAPSE score ≤18 mm, had a duration of hospital stay >10 days, whereas only 20% of patients with TAPSE score >18 mm had a duration of hospital stay >10 days (p = 0.001). The above findings suggest that patients of AMI with concomitant right ventricular dysfunction have a longer duration of hospital stay. It was observed that the all-cause mortality in AWMI patients was 17.64% as compared to 6.25% in IWMI patients. In addition, 27.9% of the AWMI cases had developed MACE during the course of hospital stay whereas only 12.5% of the IWMI cases were complicated by MACE. The above findings were in concordance with studies conducted by Stone et al. and Aher et al. and report that AWMI patients have a poorer prognosis as compared to patients of IWMI [17,15]. Another study done by Schmid et al. had also shown that patients with TAPSE score ≤18 mm (suggesting right ventricular dysfunction) had poorer outcomes as compared to patients with TAPSE score >18 mm [11]. Among patients with TAPSE score ≤18 mm, 36.23% of patients had developed MACE whereas only 12.21% of patients with TAPSE score >18 mm had reported MACE. The above findings were in accordance with a study done by Aher et al. in which they revealed that AMI patients with concomitant right ventricular dysfunction result in a higher incidence of MACE, longer duration of hospital stay, and higher mortality rate [15].

The limitation of the present study includes: (i) small sample size; (ii) we have used 2D echocardiography using TAPSE score for grading the right ventricular dysfunction; however, other modalities like tissue Doppler imaging would have helped to assess the diastolic function of the right ventricle; (iii) cardiac MRI is considered the gold standard for evaluating the right ventricular function; and (iv) lack of follow-up and thus long-term implications of the involvement of right ventricular function could not be assessed. Hence, future studies and further research are warranted on this topic on a larger scale with a higher sample size and follow-up of patients. This would pave way for a better understanding of the implications of right ventricular dysfunction on the prognosis of patients with AMI.

## Conclusions

To conclude, in the present study, it was found that AWMI results in a higher incidence of right ventricular dysfunction with a poorer prognosis as compared to IWMI. Furthermore, patients of AMI with concomitant right ventricular dysfunction were found to have poorer outcomes, in terms of longer duration of hospital stay, higher incidence of MACE, and higher mortality rate, as compared to patients of AMI without right ventricular dysfunction. This study throws light on the importance of meticulous assessment of right ventricular function in all patients of AMI, regardless of the site of infarction. Early diagnosis of right ventricular involvement is essential for prompt management of the patient.
